# A syndrome differentiation model of TCM based on multi-label deep forest using biomedical text mining

**DOI:** 10.3389/fgene.2023.1272016

**Published:** 2023-10-03

**Authors:** Lejun Gong, Jindou Jiang, Shiqi Chen, Mingming Qi

**Affiliations:** ^1^ Jiangsu Key Lab of Big Data Security and Intelligent Processing, School of Computer Science, Nanjing University of Posts and Telecommunications, Nanjing, China; ^2^ Smart Health Big Data Analysis and Location Services Engineering Lab of Jiangsu Province, Nanjing, China; ^3^ School of Data Science and Artificial Intelligence, Wenzhou University of Technology, Wenzhou, China

**Keywords:** multi-label learning, TCM syndrome differentiation, multi-label deep forest, PCC-MLRF, biomedical text mining

## Abstract

Syndrome differentiation and treatment is the basic principle of traditional Chinese medicine (TCM) to recognize and treat diseases. Accurate syndrome differentiation can provide a reliable basis for treatment, therefore, establishing a scientific intelligent syndrome differentiation method is of great significance to the modernization of TCM. With the development of biomdical text mining technology, TCM has entered the era of intelligence that based on data, and model training increasingly relies on the large-scale labeled data. However, it is difficult to form a large standard data set in the field of TCM due to the low degree of standardization of TCM data collection and the privacy protection of patients’ medical records. To solve the above problem, a multi-label deep forest model based on an improved multi-label ReliefF feature selection algorithm, ML-PRDF, is proposed to enhance the representativeness of features within the model, express the original information with fewer features, and achieve optimal classification accuracy, while alleviating the problem of high data processing cost of deep forest models and achieving effective TCM discriminative analysis under small samples. The results show that the proposed model finally outperforms other multi-label classification models in terms of multi-label evaluation criteria, and has higher accuracy in the TCM syndrome differentiation problem compared with the traditional multi-label deep forest, and the comparative study shows that the use of PCC-MLRF algorithm for feature selection can better select representative features.

## 1 Introduction

Traditional Chinese medicine (TCM) is an important part of Chinese traditional medicine with unique theoretical system and diagnostic and therapeutic methods. TCM textual data is an important carrier of TCM knowledge, which is rich and diverse, with a long history and a special language, using a large number of terminology, allusion references, metaphors and similes, *etc.*, which expresses the ideological methods and cultural connotations of TCM, but it also increases the difficulty of comprehension and analyses. TCM text data are important for studying the rules and characteristics of TCM illnesses and diseases, as well as having practical value for improving the quality and level of TCM services. Therefore, we chose TCM text data as the object of study.

At present, biomedical text mining of artificial intelligence methods are widely used in the medical field, especially in the diagnosis and treatment of diseases. But most of the research is on applications in the other biomedicine, with applications in TCM lagging behind. Different from the related research on western medicine, TCM syndrome differentiation mainly relies on doctors’ own theoretical knowledge and accumulated clinical experience. Therefore, subjective factors play a decisive role in TCM diagnosis, and this unique diagnosis mode has become a bottleneck in the development of TCM (Zhang et al., 2020; Song et al., 2021). In recent years, many domestic scholars have carried out research on standardization, digitalization and intellectualization of TCM syndrome differentiation. The main difficulties in realizing the intellectualization of TCM syndrome differentiation are as follows:

1) Syndrome is a unique concept in TCM clinical diagnosis and treatment. There are experiments show that a disease may include several different syndromes, and the same syndrome may appear in different diseases during their development. That is, to say, in TCM clinical syndrome differentiation, the syndromes tend not to appear singly, but are often intertwined and there will be two or more syndromes combined. The problem of multi-syndrome combination makes TCM syndrome differentiation essentially a typical multi-label learning problem, which makes TCM syndrome differentiation and prediction task very challenging (Zhou et al., 2018).
2) Compared with the abundant public data sets in the field of Western medicine, there is a lack of corresponding work in the TCM field. The data set of TCM is difficult to obtain and the scale is very limited. Due to the absence of TCM data collection standards, clinical data privacy protection and other reasons, it is difficult to form a large-scale standard data set in TCM (Zhang et al., 2018; Li et al., 2020; Yang and Zhu, 2021). In order to overcome this limitation, it is necessary to design a TCM syndrome differentiation analysis method suitable for small-scale data sets.
3) Generally, the data dimension of medical text is large and the value density is low, which inevitably leads to redundant and irrelevant features, affecting the performance of related classification algorithms (Shao et al., 2013; Guo et al., 2016; Huq et al., 2018). The minimum feature subset needs to be selected reasonably to maximize the performance of the model.

Numerous experts and scholars have conducted research around the problems of TCM syndrome differentiation. Xu et al. (2016) learned that in the clinical therapy of chronic gastritis, there are 30% of cases with two or more syndromes combined, so they used random forest and REAL algorithms to select the related symptoms of chronic gastritis, and finally constructed an effective model. Yang T. et al. (2020) proposed a multi-label learning algorithm for TCM syndrome differentiation based on dependency tree, which took full account of the correlation between syndromes. In this paper, we use deep forest model to deal with multi-label classification problem, and use a feature selection method based on PCC-MLRF algorithm to do multiple evidence related feature screening. Xia et al. (2020) applied multi-label K-nearest Neighbor algorithm (ML-KNN) to model 767 clinical cases and successfully established the syndrome differentiation model of metabolic syndrome. Yan et al. (2020) introduced Support Vector Machines (SVMs) into the study of TCM diagnosis and treatment, demonstrating the feasibility of using machine learning methods to approximate TCM diagnosis.

In recent years, deep neural networks (DNN) have become a new hot spot in the field of AI because of its powerful feature learning and representation capabilities. PangWeiZhao et al. (2020) combined the DNN with the attention mechanism to construct the syndrome differentiation model of 10,910 AIDS data sets, and the accuracy rate was superior to other models. However, the construction of depth learning model requires a large number of training samples, and the modulus and quality of data influence the model effect. Due to the high requirements of the amount of training data and the complicated hyper-parameter, there are certain restrictions on the application of DNN. Zhou and Feng (2017) proposed Deep Forest which is an alternative method based on deep learning ideas. Compared to DNN, Deep Forest has high efficiency and scalability, and can be used for small-scale training data tasks. Therefore, it is widely used in many fields, especially in the field of bioinformatics. LMI-DForest (Wang et al., 2020) introduced the deep forest algorithm into the prediction task of lncRNA-miRNA interactions and achieved superior performance over the other machine learning models. Chu et al. (2021) proposed a cascade deep forest model towards the prediction of drug-target interactions (DTIs) and successfully predict 1352 new DTIs which are proved to be correct. In order to find new lncRNA–protein Interactions (LPIs), Tian et al. (2021) developed a deep forest model with cascade forest structure. In view of the complexity and nonlinear characteristics of TCM, (Yan et al., 2019) used the deep forest algorithm to build a TCM syndrome classification model for chronic gastritis. Nevertheless, the above applications of Deep Forest are all single-label classification problems while multi-label learning problems emerging in large numbers (Wang et al., 2021). Recently Yang L. et al. (2020) proposed Multi-Label Deep Forest (MLDF) method, that is, the first time to introduce the deep forest model to multi-label learning tasks. MLDF is proposed with two measure-aware mechanisms: measure-aware feature reuse mechanism based on confidence computing, which can optimize different performance measures on user’s demand, and measure-aware layer growth mechanism, which can reduce overfitting.

Inspired by Yang L. et al. (2020) who proposed a MLDF approach, we first introduced MLDF to the TCM dialectic task. In order to solve the common multi-label classification problem in the TCM dialectic domain, we proposed an improved multi-label deep forest model based on the PCC-MLRF feature selection algorithm, called ML- PRDF. ML-PRDF does not simply transform TCM syndrome differentiation task into a single label classification task. In the face of complex multi-syndrome labels, the correlation of labels and the relationship between samples and multiple labels are fully considered to avoid the loss caused by label transformation. It adopted an effective feature selection algorithm to enhance the representation of features within the model, while integrating the strong representation learning ability of deep forest. This model has high computational efficiency and does not require professional knowledge in the field of TCM.

## 2 Methods

Syndrome differentiation is essentially a multi-label classification task. The syndrome differentiation analysis model of TCM inputs clinical information and outputs corresponding syndrome labels. Each instance can obtain corresponding labels through the classification model, and the label can be one or more. The TCM clinical information feature set can be expressed as $P=\left\{p_{1},p_{2},p_{3},\ldots ,p_{f}\right\}\in R^{f}$ , X stands for sample set of TCM clinical cases, $X=\left\{x_{1},x_{2},x_{3},\ldots ,x_{n}\right\}\in R^{n\times f}$ . There are n samples in the set and each sample can be represented as $x_{i}=\left(p_{i}^{1},p_{i}^{2},p_{i}^{3},\ldots ,p_{i}^{f}\right)$ , $Y\in R^{n\times m}$ represents the set of all syndrome labels that appear in the instance. $y_{i}={\left\{0,1\right\}}^{l}\in Y$ represents syndrome label corresponding to $x_{i}$ , If $y_{i}\left(l\right)=1$ , it means that the sample belongs to l label class. If $y_{i}\left(l\right)=0$ , it means that the sample does not belong to l label class.

### 2.1 Datasets

Kidney diseases and stomach diseases are the most common diseases in Chinese medicine, involving multiple systems and organs of the human body, with complex and diverse symptoms, often showing multiple symptoms corresponding to multiple symptoms, and the principle of Chinese medicine in treating these two diseases is to discriminate between different symptoms, i.e., according to the different symptoms to determine which type of symptoms they belong to, and then to treat them. Therefore, they are very suitable for TCM dialectical research.

The kidney disease dataset (Web333panda, 2021) used in this paper was obtained from the National Population Health Science Data Centre PHDA TCM Prevention and Control of Chronic Renal Failure database, and the stomach disease dataset (Web333panda, 2021) was obtained from TCM outpatient diagnosis and treatment records with necessary desensitisation. The data used in this study can be easily accessed from https://github.com/web333panda/TCM-Dataset. The data processing of disease data set is as follows: 1) Removing data records without syndromes or symptoms, 2) Combining single-label records into multi-label records.

In order to minimize the error caused by different expressions as much as possible, we established a standardized symptom dictionary based on the TCM symptom description information in the SymMap database (Wu et al., 2019). Then we merge the synonyms in the original data, transform the non-standard symptom description into standard symptom names, and reduce the feature dimension. After data integration and standardized operation, the statistics related to the dataset are shown in Table 1.

**TABLE 1:** Frequency statistics of syndromes and symptoms.

Dataset	Count	Symptom features	Max frequency/Min frequency	Syndrome labels	Max frequency/Min frequency
Kidney Disease	645	755	472/1	124	214/1
Stomach Disease	436	323	230/1	49	89/1

### 2.2 PCC-MLRF feature selection algorithm

Previously, the feature processing of multi-label classification for clinical texts mostly adopted some simple and direct strategy, such as unsupervised method or converting multi-label feature selection problems into single-label ones, which ignored the correlation among different labels (Guo et al., 2016). In multi-label learning, each instance is associated with multiple class labels, and the label information may be noisy or incomplete.

The Relief algorithms are representative algorithms in the filter method, which is the mainstream method of feature selection. ReliefF algorithm can process various types of data and has strong tolerance to noise, so it is widely used. However, ReliefF algorithm cannot be applied to multi-label learning tasks. ML-ReliefF algorithm (Cai et al., 2015) solves the problem of multi-label learning. Different from other multi-label selection methods that only consider the relationship between pairs of classes, this method introduces the concept of label set and further considers the relationship between label sets, reflecting the influence between samples and multiple labels. The similarity calculation between samples was also added to force the effect. However, the ML-ReliefF algorithm uses the cosine of the included angle of two vectors to evaluate their similarity. Cosine similarity measures the consistency of orientation between dimensions, which pays attention to the difference in direction, not numerical value. The insensitivity to numerical value causes the loss of certain information, which leads to errors in the results.

The PCC-MLRF algorithm proposed in this study improves the calculation method of sample similarity in ML-ReliefF algorithm. We use the idea of Pearson correlation coefficient (PCC) to measure the relevance of the vector, which centralize the vector before performing the cosine calculation. The error of missing information will be corrected, so PCC-MLRF is more suitable for TCM syndrome differentiation problems.

Suppose there are two samples $X=\left\{x_{1},x_{2},\ldots ,x_{n}\right\}$ and $Y=\left\{y_{1},y_{2},\ldots ,y_{n}\right\}$ , the corresponding PCC calculation formula is shown in Eq. 1. Since $\rho_{xy}$ may be negative, we use the reciprocal of Pearson distance as sample similarity, and the interval of Pearson distance is (0,2). The final sample similarity calculation formula is shown in Eq. 2.

$$\rho_{xy}=\frac{Cov\left(X,Y\right)}{\sqrt{D\left(X\right)}\sqrt{D\left(Y\right)}}=\frac{\sum_{i=1}^{n}\left(x_{i}-\bar{x}\right)\left(y_{i}-\bar{y}\right)}{\sqrt{\sum_{i=1}^{n}{\left(x_{i}-\bar{x}\right)}^{2}}\sqrt{\sum_{i=1}^{n}{\left(y_{i}-\bar{y}\right)}^{2}}}$$ (1)

$$sim=\frac{1}{1-\rho_{xy}}$$ (2)

PCC-MLRF algorithm also changes the selection method of sample point from random selection to traversal selection, avoiding the problem that high-frequency samples are repeatedly sampled while low-frequency samples are difficult to be selected. It makes the final weight update formula more reasonable.

The feature weight updating formula of PCC-MLRF algorithm is shown in Eq. 3. For a certain feature of the sample point, its initial weight minuses the feature difference of samples which have same labels with the sample point, and pluses the feature difference of samples which have different labels. The principle is that if the feature is relevant to the ability to classify, then it should be able to distinguish between samples with different labels and not confuse them, i.e., the values of similar samples should be close together and the values of dissimilar samples should be far away.

$$W_{p}=W_{p}-\sum_{j=1}^{K}{sim}_{t,{Hit}_{j}}∙\frac{d\left(p,x_{t},{Hit}_{j}\right)}{n\sum_{j=1}^{K}{sim}_{t,{Hit}_{j}}}+\sum_{C\neq C\left(x_{t}\right)}\sum_{j=1}^{K}{\frac{P\left(C\right)}{1-P\left(C\left(x_{t}\right)\right)}\cdot sim}_{t,{Miss\left(C\right)}_{j}}∙\frac{d\left(p,x_{t},{Miss\left(C\right)}_{j}\right)}{n\sum_{j=1}^{K}{sim}_{t,{Miss\left(C\right)}_{j}}}$$ (3)

Hit represents the nearest neighbors belonging to the same class as the sample point, $Miss\left(C\right)$ represents the nearest neighbors belonging to class C , $P\left(C\right)$ represents the prior probabilities of class C , $d\left(p,x_{t},{Hit}_{j}\right)$ represents the distance between sample points $x_{t}$ and ${Hit}_{j}$ with respect to feature p , ${sim}_{t,{Hit}_{j}}$ represents the sample similarity of sample points $x_{t}$ and ${Hit}_{j}$ .

The algorithm steps of PCC-MLRF feature selection algorithm are as follows:

Algorithm PCC-MLRF feature selection

**Input:**Symptom feature matrix $X=\left\{x_{1},x_{2},x_{3},\ldots ,x_{n}\right\}\in R^{n\times f}.$

Syndrome multi-label matrix $Y\in R^{n\times m}$

**Output:**List of feature indexes in descending order of weight W

**Method**:
• Initialize feature weights $W_{p}=0\left(p=1:f\right)$

• For t=1:n

• Select sample $x_{t}\text{ and }g$ et the corresponding tag set ${LS}_{t}$

• Add the top K nearest neighbors in the same sample of ${LS}_{t}$ to Hit

• For C∉ ${LS}_{t}$

Add the top K nearest neighbors in the heterogeneous sample of ${LS}_{t}$ to $Miss\left(C\right)$

• For p=1:f

• Updated feature weight $W_{p}$

End for
End for
End for
Finally get $W=argsort\left(W_{p}\right)$

### 2.3 Multi-label deep forest (MLDF)

Deep Forest is a framework based on multi-gained scanning and cascade forest, which can perform representation learning just like deep neural network model. However, unlike deep neural network, which requires large-scale training data, deep forest can work well with only small-scale training data. Multi-label Deep Forest (Yang L. et al., 2020) (MLDF) is formed by introducing deep forest into multi-label learning. Based on multi-label random forest structure and two measure-aware mechanisms, MLDF can optimize different performance measures on user’s requirements, reuse excellent representations in the previous layer, and reduce overfitting when utilizing label correlations by a large number of layers.

**FIGURE 1:**
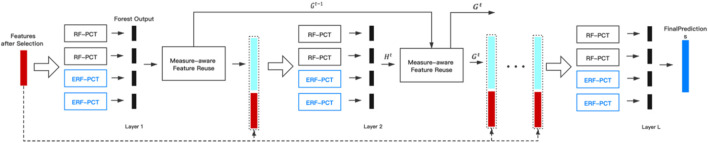
The framework of MLDF (Yang L. et al., 2020)

**FIGURE 2:**
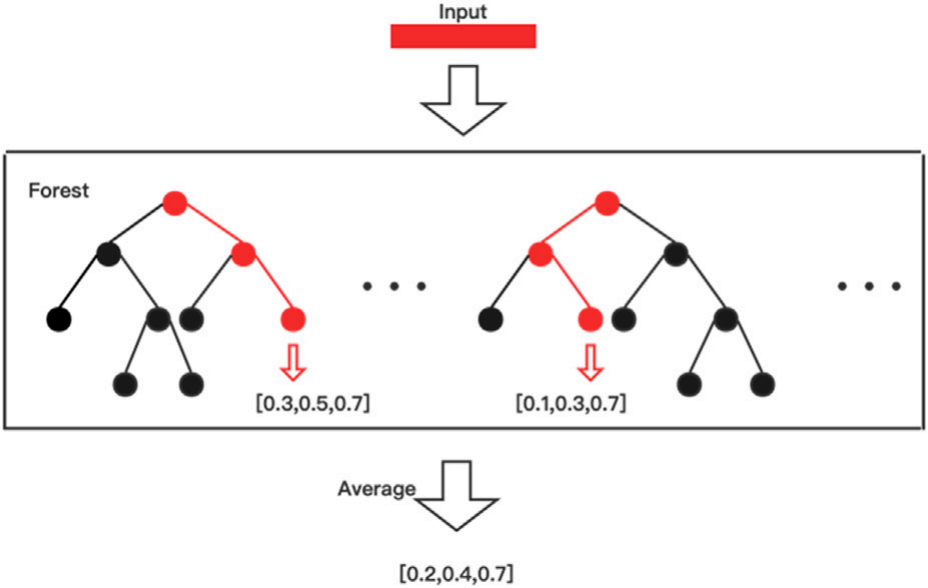
The example of a multi-label forest’s forecasting process.

The framework of MLDF is illustrated in Figure 1. With two different methods generating nodes in trees are used to forests, each layer of MLDF integrates two groups of different multi-label forests as the base classifier. RF-PCT (Kocev et al., 2013) is an algorithm that integrates random forest and predictive clustering tree. The performance of a single multi-label decision tree is limited, but it will be significantly improved after integration. For a sample, the forest gives a predicted probability vector by averaging the results for each tree. The process of multi-label forest predicting samples in the model is shown in Figure 2. The multi-label random forest averages the results of two trees to obtain the prediction label vector (0.2,0.4,0.7).

After fitting different multi-label forests ensembled in each layer, we can get an output vector $H^{t}$ . The measure-aware feature reuse mechanism will receive $H^{t}$ and update it by reusing the excellent representation of the previous layer to be $G^{t}$ . Then $G^{t}$ will be concatenated to the input feature and put into the next layer.

The measure-aware layer growth mechanism limits the complexity of the model through various measures to alleviate the overfitting problem. Based on the measures selected by the user, as long as the process is not exited, a layer will be added to the existing model. However, if the performance has not been updated in the last three layers, the layer growth process will be exited.

Table 2 lists the definition formula of six multi-label learning measures, in which the ones marked with * are label-based measures and the others are instance-based measures. “↓” indicates that the lower the value is, the better the performance is. “↑” indicates that the higher the better. n is the number of instances, m is the number of labels, $h_{ij}$ represents the prediction result of the $i^{th}$ instance on the $j^{th}$ label, $y_{ij}$ represents the corresponding real label result, $f\left(x_{i}\right)$ represents the confidence score of the instance $x_{i}$ , ${rank}_{f}\left(x_{i},j\right)$ represents the predicted score ranking of the $j^{th}$ label of the instance $x_{i}$ , $Y^{+}$ represents the label set corresponding to 1 in the real label, $Y^{-}$ represents the label set corresponding to 0 in the real label.

**TABLE 2:** Definition formula of six measures.

Measure	Formulation
*Hamming loss↓	$\frac{1}{nm}\sum_{i=1}^{n}\sum_{j=1}^{m}1\left\{h_{ij}\neq y_{ij}\right\}$
One error↓	$\frac{1}{n}\sum_{i=1}^{n}1\left\{\mathrm{argmax}\;f\left(x_{i}\right)\notin Y_{i\cdot }^{+}\right\}$
Coverage↓	$\frac{1}{nm}\sum_{i=1}^{n}\left.\left\{j\in Y_{i\cdot }^{+}\right.\right|\;\max{rank}_{f}\left(x_{i},j\right)\}-1$
Ranking loss↓	$\frac{1}{n}\sum_{i=1}^{n}\frac{\left|\left\{\left(u,v\right)\in \left(Y_{i\cdot }^{+}\times Y_{i\cdot }^{-}\right)|f_{u}\left(x_{i}\right)\leq f_{v}\left(x_{i}\right)\right\}\right|}{\left|Y_{i\cdot }^{+}\right|\left|Y_{i\cdot }^{-}\right|}$
Average precision↑	$\frac{1}{n}\sum_{i=1}^{n}\frac{1}{\left|Y_{i\cdot }^{+}\right|}\sum_{j\in Y_{i\cdot }^{+}}\frac{\left|\left\{k\in Y_{i\cdot }^{+}|{rank}_{f}\left(x_{i},k\right)\leq {rank}_{f}\left(x_{i},j\right)\right\}\right|}{{rank}_{f}\left(x_{i},j\right)}$
*Macro-AUC↑	$\frac{1}{m}\sum_{j=1}^{m}\frac{\left|\left\{\left(a,b\right)\in \left(Y_{\cdot j}^{+}\times Y_{\cdot j}^{-}\right)|f_{j}\left(x_{a}\right)\leq f_{j}\left(x_{b}\right)\right\}\right|}{\left|Y_{\cdot j}^{+}\right|\left|Y_{\cdot j}^{-}\right|}$

### 2.4 ML-PRDF: a multi-label deep forest model based on PCC-MLRF

As aforementioned in Introduction, the data dimension of medical text is large and the value density is low, which inevitably leads to redundant and irrelevant features in TCM syndrome differentiation task. Supposing that the symptom feature is directly put into the original multi-label deep forest model without feature selection, it will increase the space complexity of the algorithm, reduce the operating efficiency and finally affect the performance.

**FIGURE 3:**
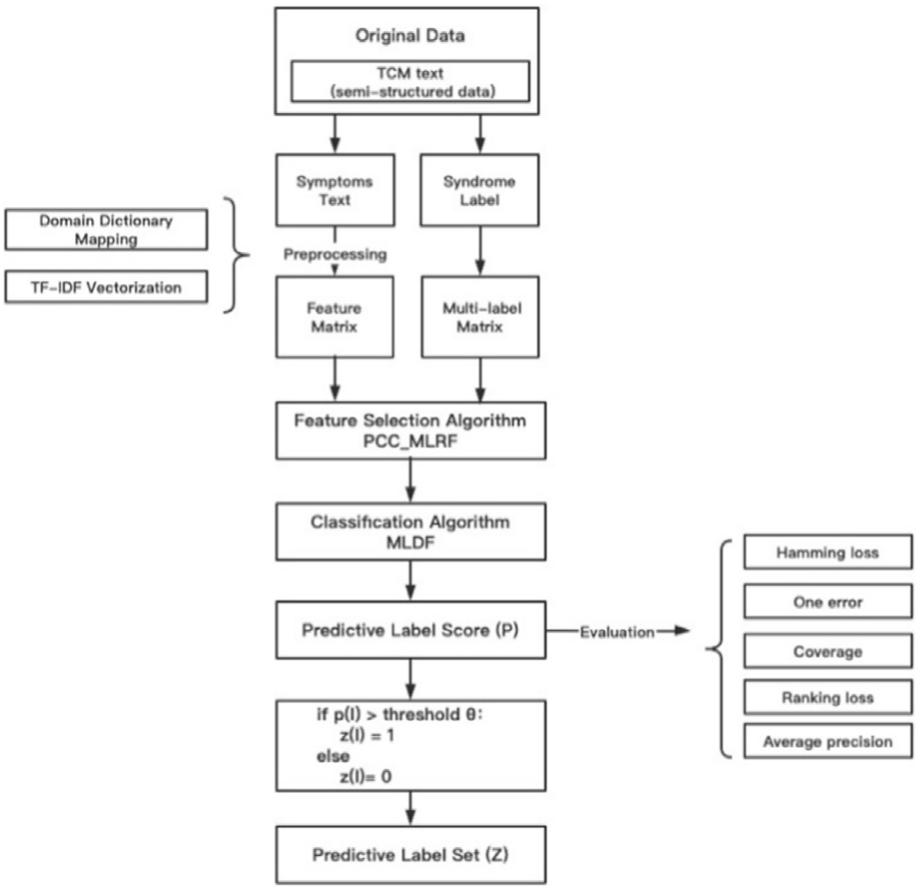
The process of ML-PRDF.

To improve the problem in the original model, this paper proposes a multi-label deep forest model based on PCC-MLRF feature selection algorithm (ML-PRDF). The predicting process of ML-PRDF can be summarized as follows. The effective experimental data were extracted from the original TCM clinical case data. After the preprocessing of removing the cases of missing symptoms or syndromes, combining single-label data into multiple-label data, TCM symptom standardized mapping and TF-IDF vectorization, we can get the input feature matrix and syndrome multi-label matrix. The PCC-MLRF feature selection algorithm was used to update the weight values of symptom features, and we will select the optimal feature subset in descending order according to the weight values. Then, multi-label deep forest was used for syndrome differentiation and prediction of TCM to obtain the score of instances for each label. If the score was higher than a certain threshold value, the sample is considered to belong to the label, and the corresponding label set of the sample will be finally obtained. Figure 3 describes the process of ML-PRDF model of TCM syndrome differentiation analysis.

## 3 Results and discussions

### 3.1 Evaluation measures

The performance of multi-label classification will be limited by redundant or unrelated features in clinical data, so it is necessary to select valid features from the feature space. Our study adopts the following five multi-label evaluation metrics based on instance to accurately evaluate the performance of the feature selection algorithm proposed in this paper from multiple aspects. These evaluation measures are defined as follows (Wang et al., 2015):

$$mlACC=\frac{1}{n}\sum_{i=1}^{n}\frac{\left|Y_{i}\cap H_{i}\right|}{\left|Y_{i}\cup H_{i}\right|}$$ (4)

$$mlPRE=\frac{1}{n}\sum_{i=1}^{n}\frac{\left|Y_{i}\cap H_{i}\right|}{\left|H_{i}\right|}$$ (5)

$$mlREC=\frac{1}{n}\sum_{i=1}^{n}\frac{\left|Y_{i}\cap H_{i}\right|}{\left|Y_{i}\right|}$$ (6)

$$mlF1=\frac{2\cdot mlREC\cdot mlPRE}{mlREC+mlPRE}$$ (7)

$$ACC=\frac{1}{n}\sum_{i=1}^{n}1\left(Y_{i}\equiv H_{i}\right)$$ (8)

$Y_{i}$ represents the real label of each instance, $H_{i}$ represents the predicted label of each instance, $\left|\cdot \right|$ represents the number of elements in the set, $1\left(Y_{i}\equiv H_{i}\right)$ means that this formula is 1 if the real label and the predicted label are exactly the same, otherwise it is 0.

### 3.2 Comparative experiments

In order to validate that the PCC-MLRF algorithm can better select representative features, we compared three classical multi-label classification algorithms on the kidney disease dataset using a feature extraction method, an unsupervised feature selection method, a traditional ML-ReliefF multi-label feature selection method and PCC-MLRF. PCA is one of the representative methods for feature extraction which can extract the main feature components of the data and variance filtering method (Remove features with low variance) was chose as the representative algorithm of unsupervised feature selection. They only extract or select features based on the distribution and variation of the data itself, without considering the correlation between the labels, which can lead to some features related to the classification ability to be ignored or lost, and therefore perform poorly, as shown in Table 3.

**TABLE 3:** Comparison of four algorithms on three classical multi-label classifiers.

Classifiers	Algorithms	mlACC	mlPRE	mlREC	mlF1	ACC
BR Boutell et al. (2004)	PCA	0.3129 ± 0.0075	0.3712 ± 0.0094	0.3759 ± 0.0072	0.3735 ± 0.0083	0.1873 ± 0.0086
	RFL	0.3085 ± 0.0071	0.3674 ± 0.0046	0.3724 ± 0.0094	0.3698 ± 0.0070	0.1839 ± 0.0068
	MLRF	**0.3345 ± 0.0103**	**0.3935 ± 0.0077**	**0.3981 ± 0.0095**	**0.3958 ± 0.0086**	**0.2096 ± 0.0120**
	PCC-MLRF	0.3281 ± 0.0056	0.3849 ± 0.0060	0.3894 ± 0.0053	0.3871 ± 0.0057	0.2062 ± 0.0051
CC Read et al. (2011)	PCA	0.3038 ± 0.0089	0.3657 ± 0.0091	0.3693 ± 0.0067	0.3675 ± 0.0079	0.1838 ± 0.0069
	RFL	0.3142 ± 0.0051	0.3776 ± 0.0042	0.3765 ± 0.0094	0.3770 ± 0.0069	0.1976 ± 0.0034
	MLRF	0.3239 ± 0.0012	0.3867 ± 0.0011	0.3891 ± 0.0020	0.3879 ± 0.0014	0.2045 ± 0.0017
	PCC-MLRF	**0.3396 ± 0.0132**	**0.4010 ± 0.0117**	**0.4042 ± 0.0152**	**0.4026 ± 0.0135**	**0.2182 ± 0.0138**
ML-KNN Zhang and ZhouKNN (2007)	PCA	0.4208 ± 0.0010	0.4807 ± 0.0150	0.4836 ± 0.0060	0.4820 ± 0.0046	0.2956 ± 0.0172
	RFL	0.3676 ± 0.0113	0.4286 ± 0.0074	0.4332 ± 0.0126	0.4308 ± 0.0077	0.2406 ± 0.0222
	MLRF	0.4125 ± 0.0068	0.4747 ± 0.0065	0.4744 ± 0.0075	0.4745 ± 0.0023	0.2904 ± 0.0137
	PCC-MLRF	**0.4371 ± 0.0131**	**0.4997 ± 0.0030**	**0.4976 ± 0.0152**	**0.4986 ± 0.0091**	**0.3144 ± 0.0207**

The meaning of bold displays better performance.

It can be seen from the above results that PCC-MLRF feature selection method has the best performance on the classification algorithms that consider the relevance between labels. It indicates that PCC-MLRF feature selection method fully learns the correlation between labels and labels as well as the correlation between labels and features, and enhances the representativeness of features within the model.

**FIGURE 4:**
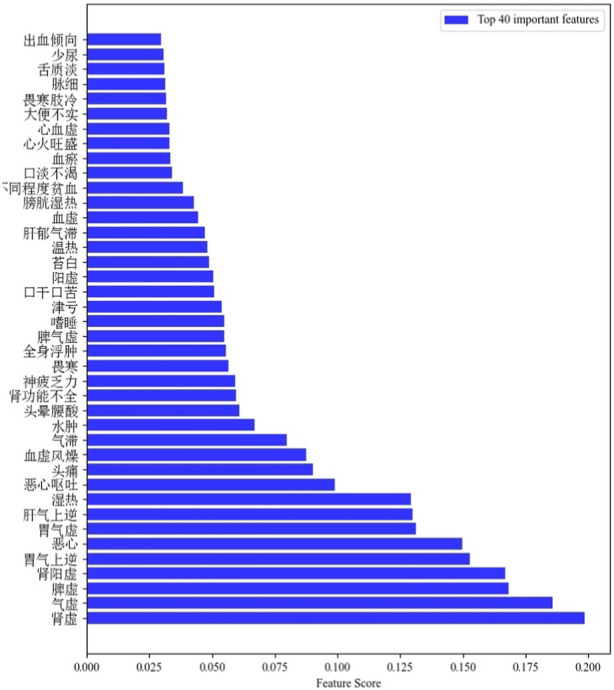
Top 40 important features.

The PCC-MLRF algorithm was used to update the weight of symptom features to obtain the importance of each feature for classification ability. Figure 4 shows examples of the 40 features with the highest weight values.

Meanwhile, in order to verify the effectiveness of ML-PRDF model in solving TCM syndrome differentiation problem, we select three classical multi-label classification algorithms for comparison, including Label Powerset (Madjarov et al., 2012), ML-KNN (Zhang and ZhouKNN, 2007), BP-MLL (Zhang and Zhou, 2006). LP is problem transformation method, which converts multi-label problems into multiple classification problems. ML-KNN is an algorithmic adaptive method, which extends specific learning algorithms in single-label problem to handle multi-label data directly. BP-MLL is a neural network algorithm derived from back propagation. All the following algorithms use the results of 5-fold cross-validation for statistical analysis.

From the perspective of clinical TCM syndrome differentiation in Table 4, hamming loss reflects the misjudgment rate of the syndrome differentiation results. One error reflects the error rate of the model in searching for the most relevant syndromes. Coverage reflects the redundancy rate of unrelated syndromes in the predicted results. Ranking loss reflects the proportion of inverted order label pairs, that the smaller the value is, the higher the similarity between the predicted label ranking and the real ranking is. Average precision reflects the similarity between the predicted results and the real results.

**TABLE 4:** Comparison of different classification models for kidney disease datasets.

Classifiers	Hamming loss↓	One error↓	Coverage↓	Ranking loss↓	Average precision↑
Label Powerset	0.0127 ± 0.0002	**0.5944 ± 0.0185**	0.1714 ± 0.0020	0.2285 ± 0.0163	0.4182 ± 0.0125
ML-KNN	0.0122 ± 0.0003	0.6254 ± 0.0031	0.1748 ± 0.0092	0.1714 ± 0.0126	0.4379 ± 0.0099
BP-MLL	0.0153 ± 0.0003	0.6636 ± 0.0052	0.1887 ± 0.0102	0.1676 ± 0.0096	0.4471 ± 0.0091
ML-PRDF	**0.0102 ± 0.0004**	0.6367 ± 0.0125	**0.1540 ± 0.0034**	**0.1506 ± 0.0073**	**0.5168 ± 0.0085**

The meaning of bold displays better performance.

Compared with other multi-label learning algorithms (Table 5), ML-PRDF performs best in hamming loss, coverage, ranking loss and average precision. The predicted results of the model are highly consistent with the real syndrome differentiation results, and redundant syndrome results are few.

**TABLE 5:** Comparison of different classification models for stomach disease datasets.

Classifiers	Hamming loss↓	One error↓	Coverage↓	Ranking loss↓	Average precision↑
Label Powerset	0.0120 ± 0.0002	0.6104 ± 0.0175	0.1614 ± 0.0032	0.2125 ± 0.093	0.4976 ± 0.0045
ML-KNN	0.0132 ± 0.0002	0.6054 ± 0.0012	0.1621 ± 0.0078	0.1921 ± 0.0016	0.5179 ± 0.0019
BP-MLL	0.0134 ± 0.0003	0.6426 ± 0.0032	0.1797 ± 0.0121	0.1976 ± 0.0078	0.4821 ± 0.0102
ML-PRDF	**0.0093 ± 0.0003**	**0.4613 ± 0.0075**	**0.1507 ± 0.0074**	**0.1890 ± 0.0026**	**0.6014 ± 0.0105**

The meaning of bold displays better performance.

**FIGURE 5:**
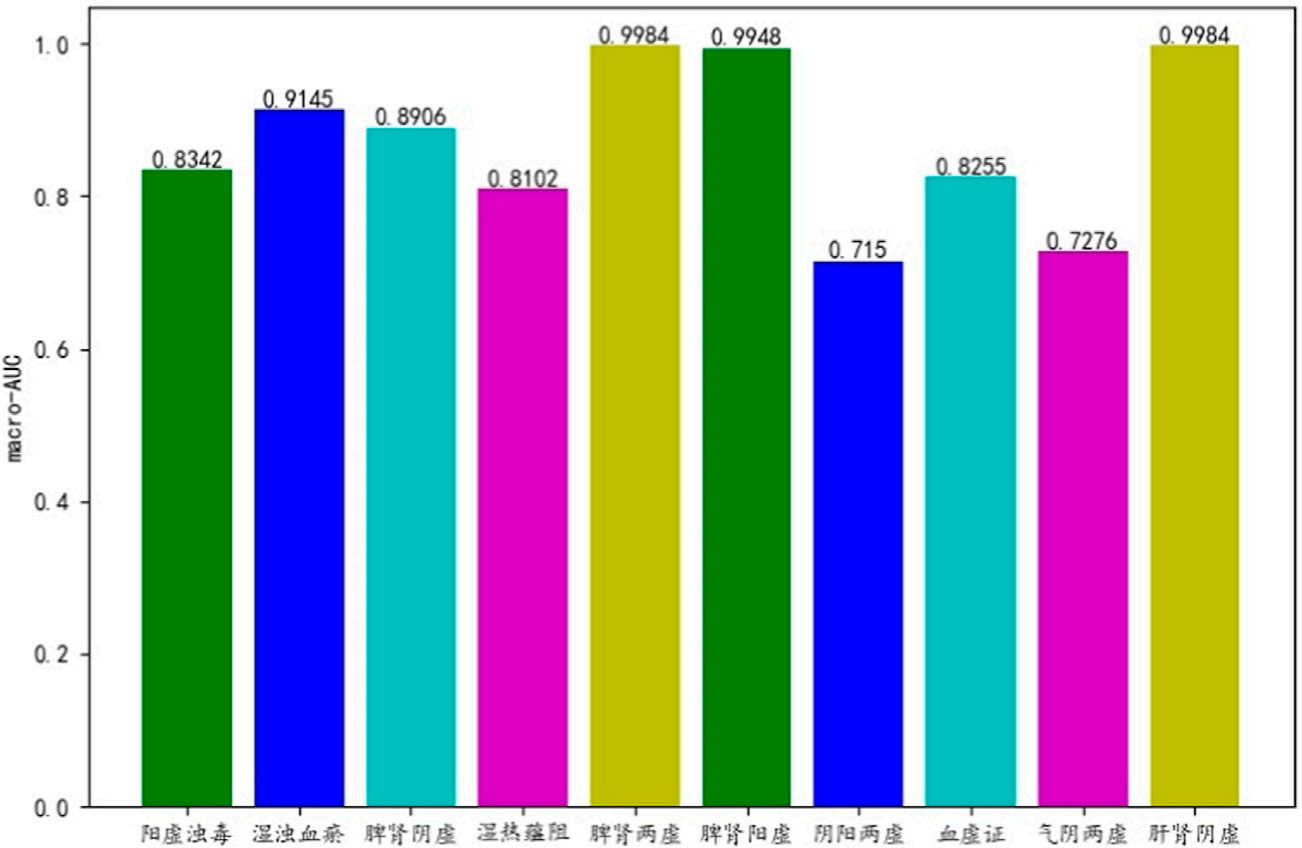
AUC of ML-PRDF in 10 syndrome samples.

Figure 5 shows the AUC value of 10 syndromes differentiating by ML-PRDF. It can be seen that the model has high classification accuracy for the common syndromes of chronic renal failure (CRF), such as damp turbidity and blood stasis, spleen-kidney yang deficiency, spleen-kidney yin deficiency and liver-kidney yin deficiency (Chen et al., 2019; Tian and Fan, 2019). It also has relatively good performance for other low-frequency syndrome labels.

To further validate the multi-label classification performance of ML-PRDF, we conducted experiments on stomach disease data with the following results:

From the above Table 5, it can be seen that the performance of ML-PRDF is optimal in all aspects, especially in Hamming loss, One error, Average precision is greatly improved compared with the other three models, which proves that ML-PRDF has strong multi-label classification performance.

### 3.3 Ablation experiments

In order to verify the necessity and effectiveness of the PCC-MLRF feature selection algorithm to improve the performance of TCM evidence typing analysis models, we designed ablation experiments. According to the evaluation measures in Table 2, the MLDF model with the removal of PCC-MLRF is compared with the ML-PRDF model with the addition of PCC-MLRF, and the experimental results are as follows:

As can be seen from the results of ablation experiment in Table 6, after adding the PCC-MLRF feature selection algorithm to the multi-label deep forest model, all metrics are significantly improved. This method effectively screens out the optimal feature subset, removes redundant and irrelevant symptom features, and reduces classification errors.

**TABLE 6:** Comparison of ablation experiments.

Dataset	Classifiers	Hamming loss↓	One error↓	Coverage↓	Ranking loss↓	Average precision↑
Kidney Disease	MLDF	0.0105 ± 0.0002	0.6480 ± 0.0166	0.1718 ± 0.0041	0.1647 ± 0.0048	0.4884 ± 0.0065
	ML-PRDF	**0.0102 ± 0.0004**	**0.6367 ± 0.0125**	**0.1540 ± 0.0034**	**0.1506 ± 0.0073**	**0.5168 ± 0.0085**
Stomach Disease	MLDF	0.0106 ± 0.0003	0.5912 ± 0.0026	0.1986 ± 0.0124	0.2258 ± 0.0085	0.5023 ± 0.0130
	ML-PRDF	**0.0093 ± 0.0003**	**0.4613 ± 0.0075**	**0.1507 ± 0.0074**	**0.1890 ± 0.0026**	**0.6014 ± 0.0105**

The meaning of bold displays better performance.

In conclusion, ML-PRDF model has the best classification performance in hamming loss, coverage, ranking loss and average precision, and also has considerable performance on the classification of a single syndrome. The fusion of PCC-MLRF and MLDF enables the correlation between symptoms and symptoms as well as symptoms and syndromes to be fully explored, and then a TCM syndrome classifier with good performance is constructed.

## 4 Conclusion

The main contributions of this paper are summarized as follows:

1) According to the characteristics of TCM clinical text, we improve the MLRF feature selection algorithm, changing the calculation method of sample similarity that only focuses on the consistency of direction to the method that focuses on both numerical values, and finally obtain PCC-MLRF.
2) We introduce the multi-label deep forest model into the field of TCM for the first time, using the powerful representation learning capabilities of deep forest.
3) Considering feature redundancy and poor operation efficiency problems of the original MLDF model, combined with the difficulties caused by the multicollinearity problem of TCM syndrome differentiation task, we propose a new model (ML-PRDF), which is an improved multi-label deep forest model based on PCC-MLRF feature selection algorithm.
4) Our experiments show that the PCC-MLRF algorithm does have better performance in the classification algorithm considering label correlation. In the meantime, compared with several multi-label classification models, ML-PRDF also achieves the best performance.
5) ML-PRDF can solve the problems of multi-syndrome combination, small-scale and non-standard TCM data set, and redundant or irrelevant features in the data. It takes full account of the correlation between labels, enhances the representativeness of features within the model, expresses the original information with fewer features, and effectively improves the performance of syndrome differentiation. Our work can provide useful reference for the development of TCM multi-label syndrome differentiation task and provide a certain auxiliary role for TCM syndrome differentiation and treatment.

Due to the complexity of the source of the experimental data set, the experiment in this paper has certain limitations. If the follow-up case texts can be collected in a standardized manner according to certain standards, and the main syndromes and secondary syndromes can be distinguished and classified, it should improve the results of TCM syndrome differentiation experiment.

## Data Availability

Publicly available datasets were analyzed in this study. This data can be found here: https://www.ncmi.cn/index.htm and https://github.com/web333panda/TCM-Dataset.
